# Antipsychotic- and Anxiolytic-like Properties of a Multimodal Compound JJGW08 in Rodents

**DOI:** 10.3390/ijms232415929

**Published:** 2022-12-14

**Authors:** Elżbieta Żmudzka, Klaudia Lustyk, Monika Głuch-Lutwin, Barbara Mordyl, Alicja Zakrzewska-Sito, Paweł Mierzejewski, Jolanta Jaśkowska, Marcin Kołaczkowski, Jacek Sapa, Karolina Pytka

**Affiliations:** 1 Department of Social Pharmacy, Faculty of Pharmacy, Jagiellonian University Medical College, Medyczna 9, 30-688 Krakow, Poland; 2 Department of Pharmacodynamics, Faculty of Pharmacy, Jagiellonian University Medical College, Medyczna 9, 30-688 Krakow, Poland; 3 Department of Pharmacobiology, Faculty of Pharmacy, Jagiellonian University Medical College, Medyczna 9, 30-688 Krakow, Poland; 4 Department of Pharmacology, Institute of Psychiatry and Neurology, Sobieskiego 9, 02-957 Warszawa, Poland; 5 Department of Organic Chemistry and Technology, Faculty of Chemical and Engineering and Technology, Cracow University of Technology, Warszawska 24, 31-155 Krakow, Poland; 6 Department of Medicinal Chemistry, Faculty of Pharmacy, Jagiellonian University Medical College, Medyczna 9, 30-688 Krakow, Poland

**Keywords:** antipsychotic-like activity, anxiolytic-like effect, D_2_ receptor antagonist, 5-HT_1A_ receptor antagonist, arylpiperazine derivative of salicylamide

## Abstract

Schizophrenia is a chronic mental illness, which remains difficult to treat. A high resistance to the available therapies, their insufficient efficacy, and numerous side effects are the reasons why there is an urgent need to develop new antipsychotics. This study aimed to assess the antipsychotic-like effects of JJGW08, a novel arylpiperazine alkyl derivative of salicylamide, in rodents. First, considering the JJGW08 receptor profile, we investigated the compound’s intrinsic activity towards dopamine D_2_ and serotonin 5-HT_1A_, 5-HT_2A_, and 5-HT_7_ receptors using functional assays. Next, we assessed the effect of JJGW08 on MK-801- and amphetamine-induced hyperlocomotion, its risk of inducing catalepsy and impairing motor coordination, as well as the anxiolytic-like effects in the four-plate and marble burying tests in mice. Finally, we investigated the antipsychotic-like properties of JJGW08 in rats using MK-801-induced hyperlocomotion and prepulse inhibition tests. We found that JJGW08 showed antagonistic properties at dopamine D_2_ and serotonin 5-HT_1A_, 5-HT_2A_, and 5-HT_7_ receptors. However, the effect on the 5-HT_2A_ and 5-HT_7_ receptors was very weak. Moreover, the tested compound showed an antipsychotic-like effect in MK-801- and amphetamine-induced hyperlocomotion but not in a prepulse inhibition test in rats. Notably, JJGW08 demonstrated anxiolytic-like properties in both behavioral tests. Importantly, the compound did not induce catalepsy or motor coordination impairment in mice at antipsychotic-like doses. Our study suggests it is worth searching for new potential antipsychotics among arylpiperazine alkyl derivatives of salicylamide.

## 1. Introduction

Schizophrenia, a chronic psychiatric disorder, affects approximately 24 million people worldwide [1]. Psychotic and motivational symptoms of the disease, accompanied by anxiety and cognitive impairments, strongly interfere with normal functioning in society [2]. Patients with schizophrenia struggle to have stable relationships, work a full-time job, be fully independent, and are more vulnerable to addictions [3]. Moreover, the available treatment strategies are neither fully effective, relieving mainly psychotic symptoms, nor entirely safe to use [4]. Therefore, there is an urgent need to develop novel antipsychotics.

Numerous studies indicated the role of the dopaminergic and serotonergic systems in schizophrenia and its treatment [5,6]. Excessive dopamine transmission in the mesolimbic pathway causes psychotic symptoms of the disease, whereas a low level of dopamine in the mesocortical pathway leads to negative and cognitive symptoms [7]. In contrast, the activation of 5-HT_2A_ receptors acts psychoactive and induces hallucination. Furthermore, Meltzer et al. proved that the blockade of serotonin receptors results in an antipsychotic effect [8]. Accordingly, atypical antipsychotic drugs produce an extensive blockade of 5-HT_2A_ receptors and reduce D_2_-mediated neurotransmission [9]. 

Scientists discovered that arylpiperazine derivatives show significant antipsychotic properties in rodents [10,11,12]. This effect is possibly mediated by D_2_ and 5-HT_2A_ receptor blockade, as these derivatives bind strongly to dopamine and serotonin receptors [13,14,15]. Moreover, compounds with 2-metoxyphenylpiperazine fragment often target 5-HT_1A_ and 5-HT_7_ receptors [16,17]. Such compounds frequently show anxiolytic-like properties. Therefore, encouraged by the above findings, we aimed to determine the antipsychotic and anxiolytic-like properties of JJGW08, a novel arylpiperazine alkyl derivative of salicylamide with high affinity for D_2_ and 5-HT_1A_ and moderate for the 5-HT_2A_ and 5-HT_7_ receptors [18,19]. First, we investigated the compound’s intrinsic activity towards dopamine D_2_ and serotonin 5-HT_1A_, 5-HT_2A_, and 5-HT_7_ receptors. Next, we assessed the antipsychotic-like properties of the compound in rats and mice. We also determined an anxiolytic potential of JJGW08 in mice. Finally, we evaluated the risk of JJGW08 to induce catalepsy or motor coordination impairments in mice.

## 2. Results

### 2.1. JJGW08 Showed Antagonistic Properties at 5-HT_1A_, 5-HT_2A_, 5-HT_7_, and D_2_ Receptors

Based on the JJGW08 receptor profile, as the first step, we evaluated its intrinsic activity towards selected receptors in functional assays. We used serotonin or α-methylserotonin as the reference agonist for 5-HT_1A_, 5-HT_2A_, and 5-HT_7_ receptors, whereas NAN-190, mianserin, and SB-269970 were used as reference antagonists, respectively. In the case of D_2_ receptors in agonist mode, we used quiniprole and apomorphine, while in antagonist mode, chlorpromazine was used as a reference ligand.

In functional assays, JJGW08 demonstrated strong antagonistic properties towards dopamine D_2_ and serotonin 5-HT_1A_ receptors and very weak antagonistic properties at serotonin 5-HT_2A_ and 5-HT_7_ receptors (Table 1). In all cases, the effect was weaker than that for reference compounds, i.e., NAN-190, mianserin, SB-269970, and chlorpromazine. Additional data are presented in Supplementary Materials (Figures S1–S4).

### 2.2. JJGW08 Reversed MK-801- and Amphetamine-Induced Hyperlocomotion in Mice

Since JJGW08 showed antagonistic properties towards D_2_ receptors, we next evaluated its antipsychotic-like properties in MK-801- and amphetamine-induced hyperlocomotion tests in mice. Compounds that decrease the hyperactivity of animals in these tests may possess antipsychotic-like properties.

**Figure 1:**
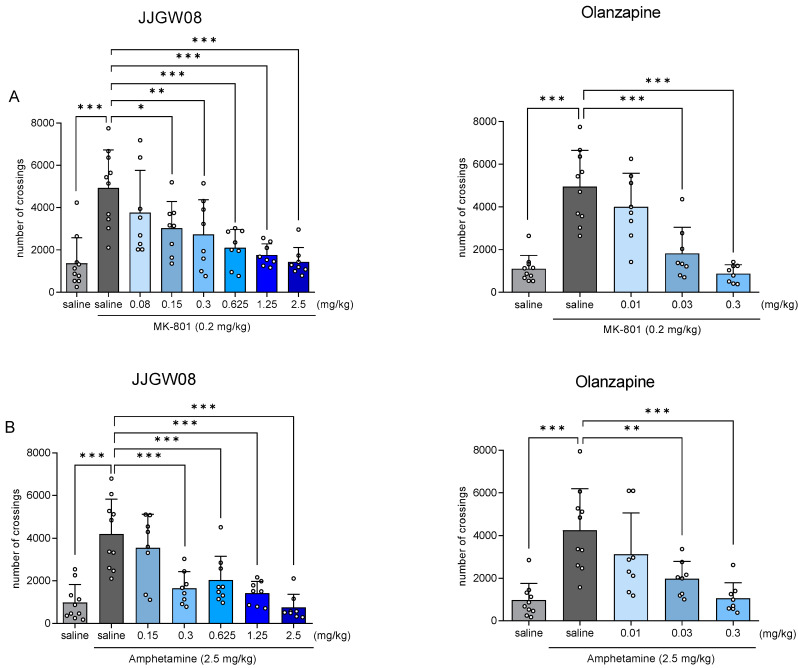
The effect of JJGW08 and olanzapine on the MK-801- (Panel (**A**)) and amphetamine-induced (Panel (**B**)) hyperlocomotion in mice. Locomotor activity was recorded in actometers separately for each mouse. After 30 min of adaptation, the number of crossings of photo beams was measured during 60 min. JJGW08 and olanzapine were administered intraperitoneally (*ip*) 30 min before the test. MK-801 (0.2 mg/kg, *ip*) was administered 15 min before the experiment, while amphetamine (2.5 mg/kg) was administered subcutaneously (*sc*) 30 min before the experiment. The control group received two injections of 0.9% NaCl (*ip* or *sc*) or 0.9% NaCl and 1% Tween in the case of olanzapine. Values are expressed as means ± SD, n = 8–10 mice per group. Statistical analysis: one-way ANOVA (Newman–Keuls *post hoc*), * *p* < 0.05, ** *p* < 0.01, *** *p* < 0.001.

JJGW08 reduced the hyperactivity of animals induced by MK-801 at the doses of 0.15–2.5 mg/kg by 24–71% at the significance level 0.05 (one-way ANOVA: F(7,60) = 7.729, *p* < 0.0001). Olanzapine, used as the reference compound, reversed hyperlocomotion at the doses of 0.03 and 0.3 mg/kg by 63 and 82%, respectively (one-way ANOVA: F(4,39) = 20.240, *p* < 0.0001) (Figure 1A).

Moreover, JJGW08 significantly reduced amphetamine-induced hyperlocomotion in mice at the range of doses of 0.3–2.5 mg/kg by 52–82% (one-way ANOVA: F(6,54) = 12.570, *p* < 0.0001). Olanzapine reduced the effect of the administered amphetamine at the doses of 0.03 and 0.3 mg/kg by 54 and 75%, respectively (one-way ANOVA: F(4,39) = 9.788, *p* < 0.0001) (Figure 1B).

### 2.3. JJGW08 Did Not Induce Catalepsy in the Bar Test in Mice at Antipsychotic-like Doses

Knowing that D_2_ receptor antagonists may induce catalepsy in animals, next, we evaluated the risk of the cataleptogenic effect of JJGW08 in the bar test in mice. Catalepsy in rodents manifests as an imposed posture for a prolonged period.

The minimum cataleptogenic dose was defined as the lowest dose inducing a mean catalepsy score of ≥1 at 30, 60, or 120 min post-treatment [21].

The lowest cataleptogenic dose of JJGW08 in the bar test in mice was 10 mg/kg. Similarly, the minimum cataleptogenic dose of olanzapine, used as the reference compound, was 10 mg/kg (Table 2).

### 2.4. JJGW08 Did Not Influence the Motor Coordination in Mice

Compounds acting within the central nervous system may disturb motor coordination. Therefore, the next step of our studies was to determine the effect of JJGW08 on motor coordination in the rotarod test in mice. The inability of rodents to remain on the rotating rod indicates the risk of the tested compound to impair motor coordination.

JJGW08 did not affect motor coordination in mice. The number of animals that fell from the rotating rod, time before animals fell, and the TD_50_ values are presented in Table 3.

### 2.5. JJGW08 Increased the Number of Punished Crossings in Four-Plate Test in Mice

The blockade of 5-HT_1A_ and 5-HT_7_ receptors may result in an anxiolytic-like effect. Hence, as the next step of our studies, we assessed the anxiolytic-like activity of JJGW08 in the four-plate test in mice. Compounds with anxiolytic-like properties increase the number of punished crossings in mice. 

**Figure 2:**
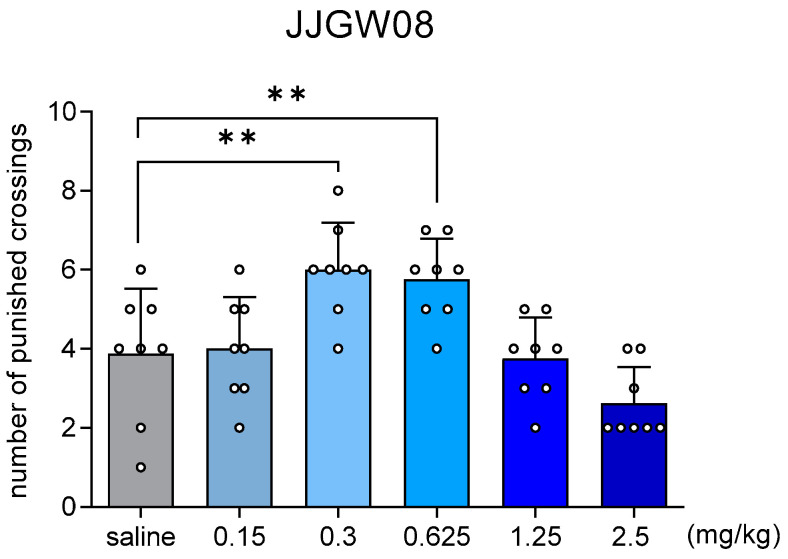
The effect of JJGW08 on the number of punished crossings in the four-plate test. Mice were placed in the apparatus, and after a 15 s adaptation period, each mouse crossing from one plate to another was punished with an electric shock. JJGW08 was administered intraperitoneally (*ip*) 30 min before the test. The control group received 0.9% NaCl solution (*ip*). Values are expressed as means ± SD, n = 8–10 mice per group. Statistical analysis: one-way ANOVA (Newman–Keuls *post hoc*), ** *p* < 0.01.

JJGW08 at the doses of 0.3 and 0.625 mg/kg increased the number of punished crossings by 54 and 49%, respectively (one-way ANOVA: F(5,42) = 9.103, *p* < 0.0001) (Figure 2).

### 2.6. JJGW08 Decreased the Number of Buried Marbles by Mice

In order to confirm the anxiolytic-like properties of JJGW08, we decided to perform the marble burying test, which is also dedicated to assess anxiolytic-like activity. The reduction of the buried marbles number indicates an anxiolytic-like effect of the tested compound. 

**Figure 3:**
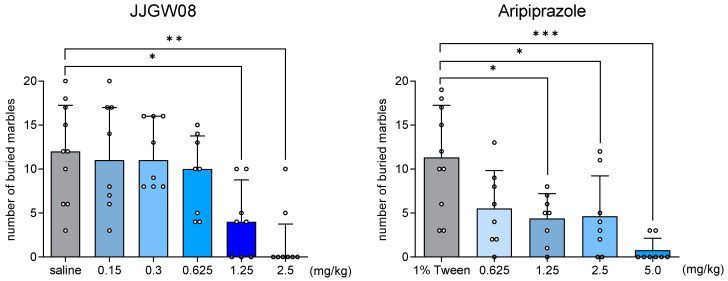
The effect of JJGW08 and aripiprazole on the number of buried marbles by mice. Mice were placed individually in cages with a 5 cm bedding layer, where 20 glass balls were placed. The number of buried marbles after 30 min of the test was counted. JJGW08 was administered intraperitoneally (*ip*) 30 min before the test. The control group received 0.9% NaCl solution (*ip*) or 1% Tween (*ip*) for aripiprazole. Values are expressed as means ± SD in the case of one-way ANOVA (aripiprazole) or median with interquartile range in the case of Kruskal–Wallis test (JJGW08), n = 8–10 mice per group. Statistical analysis: one-way ANOVA (Newman–Keuls *post hoc*), and Kruskal–Wallis test (Dunn *post hoc*), * *p* < 0.05, ** *p* < 0.01, *** *p* < 0.001.

JJGW08 significantly reduced the number of buried marbles by 65 and 84% at the doses of 1.25 and 2.5 mg/kg (Kruskal–Wallis test: H(6,50) = 20.77, *p* < 0.001) (Figure 3). In contrast, aripiprazole decreased this number at the doses 1.25, 2.5, and 5.0 mg/kg by 61, 69, and 93%, respectively (one-way ANOVA: F(4,37) = 7.363, *p* < 0.001) (Figure 3).

### 2.7. JJGW08 Did Not Influence Locomotor Activity in Mice

To verify if the results obtained in behavioral tests in mice are specific to the antipsychotic-like effect, we investigated the influence of JJGW08 on the spontaneous locomotor activity in mice. Changes in the locomotor activity of animals may suggest potential sedative or psychostimulant properties of the tested compound.

JJGW08 did not affect locomotor activity in mice in the 60-min session (one-way ANOVA: F(5,43) = 0.560, *p* = 0.730), 30-min session (one-way ANOVA: F(5,42) = 1.645, *p* = 0.169) (Table 4). The one-way ANOVA showed a significant effect in the 1-min session (one-way ANOVA: F(5,44) = 3.446, *p* < 0.05), but *post hoc* analysis revealed no differences between the studied groups (Table 4).

### 2.8. JJGW08 Reduced MK-801-Induced Hyperlocomotion in the Open Field in Rats

As the next step of our study, we verified the results of hyperlocomotion tests in mice using different species. Therefore, we performed the hyperlocomotion test induced by MK-801 in the open field in rats. Compounds with potential antipsychotic-like properties decrease locomotor hyperactivity.

**Figure 4:**
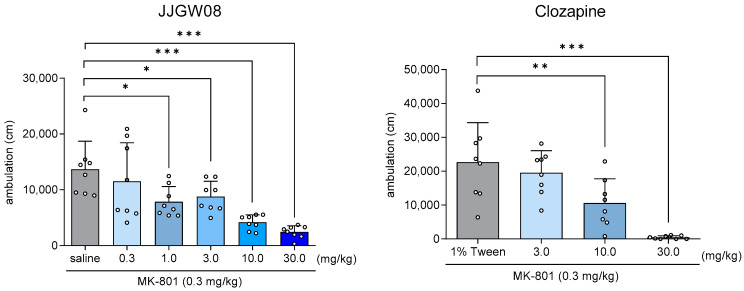
The effect of JJGW08 and clozapine on MK-801-induced hyperlocomotion in rats. After the habituation period (30 min, 24 h before the test), locomotor activity was measured individually for each animal in the special apparatus. The walked distance (cm) was monitored at 10-min intervals for 30 min, using a computerized system that tracked the animals’ movement and behavior. JJGW08 and clozapine were administered intraperitoneally (*ip*) 30 or 60 min before the test, respectively, while MK-801 (0.3 mg/kg) was administered *ip* 15 min before the start of the experiment. The control group received 0.9% NaCl solution (*ip*) (or 1% Tween (*ip*) for clozapine) and MK-801 (0.3 mg/kg, *ip*). Values are expressed as mean ± SD, n = 7–8 rats per group. Statistical analysis: one-way ANOVA (Newman–Keuls *post hoc*) * *p* < 0.05, ** *p* < 0.01, *** *p* < 0.001.

JJGW08 significantly reduced MK-801-induced hyperlocomotion in the open field by 36–82% at the doses of 1–30 mg/kg (one-way ANOVA: F(5,42) = 9.591, *p* < 0.001), whereas clozapine reduced the hyperlocomotion at the doses 10 and 30 mg/kg by 53 and 98%, respectively one-way ANOVA: (F(3,28) = 13.830, *p* < 0.0001) (Figure 4).

### 2.9. JJGW08 Did Not Reverse Deficits in Sensorimotor Gating Induced by MK-801 in Rats

To verify the effects obtained in the hyperlocomotion test in rats, we decided to perform one more test—the prepulse inhibition test in rats. The administration of stimuli (prepuls-and-pulse trials: 84/120–prepuls 84 dB (20 ms), pulse 120 dB (40 ms); 90/120–prepuls 90 dB (20 ms), pulse 120 dB (40 ms), sham stimulus (70 dB, 40 ms)), and response recording were controlled during the experiment. Reversing the deficits in sensorimotor gating induced by MK-801 suggests the potential antipsychotic-like activity of the studied compound.

**Figure 5:**
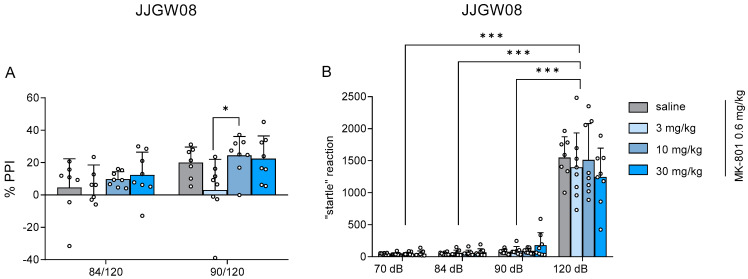
The influence of JJGW08 on startle response inhibition (Panel (**A**)) and changes in startle response (Panel (**B**)) in prepulse inhibition test after MK-801 administration in rats. The test was conducted in the soundproof apparatus, and the startle response amplitude was recorded. The experimental session (after a 5-min habituation period) had strictly defined parameters (prepuls-and-pulse trials: 84/120–prepuls 84 dB (20 ms), pulse 120 dB (40 ms); 90/120–prepuls 90 dB (20 ms), pulse 120 dB (40 ms), sham stimulus (70 dB, 40 ms)), and the startle intensity for each stimulus used in the session was measured. JJGW08 was administered *ip* 30 min before the test, while MK-801 (0.6 mg/kg) was administered *ip* 15 min before the start of the experiment. The control group received 0.9% NaCl solution (*ip*) and MK-801 (0.6 mg/kg, *ip*). Values are expressed as mean ± SD, n = 7–8 rats per group. Statistical analysis: two-way ANOVA with repeated measures (Bonferroni *post hoc*), * *p* < 0.05, *** *p* < 0.001, %PPI—a percentage of prepulse inhibition. The magnitude of the startle response is presented in arbitrary manufacturer’s units.

JJGW08 did not reverse the deficits in sensorimotor gating induced by MK-801 and presented the startle reaction inhibition at the range of −0.2 to 24.4%. Statistical analysis showed a significant prepulse inhibition effect (two-way ANOVA: F(1,27) = 17.610, *p* < 0.001) but no significant influence of the compound (two-way ANOVA: F(3,27) = 2.839, *p* = 0.057) and no interaction (two-way ANOVA: F(3,27) = 1.159, *p* = 0.344). Moreover, post hoc analysis detected significant differences between the two studied groups at 90 dB (3.0 mg/kg *vs.* 10 mg/kg; *p* < 0.05) (Figure 5A).

JJGW08 significantly increased the startle reaction amplitude in the treatment group at 120 dB. Statistical analysis for JJGW08 showed a significant startle reaction effect (two-way ANOVA: F(3,27) = 233.100, *p* < 0.001) but no significant influence of the compound (two-way ANOVA: F(3,27) = 0.234, *p* = 0.872) and no interaction (two-way ANOVA: F(9,27) = 0.787, *p* = 0.629). The *post hoc* analysis detected significant differences between all tested groups (70 dB, 84 dB, 90 dB) and the 120 dB group (Figure 5B).

## 3. Discussion

We found that JJGW08 showed moderate antagonistic properties at dopamine D_2_ and serotonin 5-HT_1A_, weak at 5-HT_7_, and very weak at 5-HT_2A_ receptors. Moreover, the tested compound demonstrated an antipsychotic-like effect in MK-801- and amphetamine-induced hyperlocomotion tests in mice. The antipsychotic-like effect was confirmed in rats. Notably, JJGW08 showed anxiolytic-like properties in mice in two behavioral tests. Finally, the compound did not induce catalepsy or motor coordination impairments at antipsychotic-like doses in mice.

The D_2_ and 5-HT_2A_ receptors play a significant role in schizophrenia and the antipsychotic effect [23,24]. Therefore, drugs targeting these receptors are used in the treatment of psychosis. Many compounds with 2-methoxyphenylpiperazine fragment show a high affinity for the above receptors [17,25]. Moreover, compounds with 2-metoxyphenylpiperazine fragment often target 5-HT_1A_ and 5-HT_7_ receptors [16,17], showing anxiolytic-like properties. Based on the receptor profile of JJGW08, which showed a high affinity for the D_2_ and 5-HT_1A_ receptors, and a moderate affinity for the 5-HT_2A_ and 5-HT_7_ receptors [19], as the first step of our studies, we evaluated its intrinsic activity towards these receptors. Our functional studies revealed that JJGW08 acted as a moderate antagonist at D_2_ and 5-HT_1A_ receptors, and a weak antagonist at the 5-HT_7_, and very weak at the 5-HT_2A_ receptor. 

Since D_2_ receptors play a pivotal role in schizophrenia, we next assessed the antipsychotic-like properties of the compound using an MK-801-induced hyperlocomotion test in mice. MK-801 blocks NMDA receptors non-competitively and, by stimulating the activity of most dopaminergic neurons in the mesolimbic region, increases dopamine release in the striatum, medial prefrontal cortex, and nucleus accumbens [26,27,28]. As a result, an antagonist of NMDA receptors causes hyperlocomotion, circling, as well as social withdrawal, mimicking both positive and negative symptoms of schizophrenia [29,30]. The ability of drug candidates to reverse MK-801-induced hyperlocomotion indicates potential antipsychotic properties. Our experiments revealed that JJGW08 significantly reduced the increased locomotor activity in mice in a wide dose range (0.15–2.5 mg/kg). Given the promising results, we next investigated the antipsychotic-like effect of JJGW08 in amphetamine-induced hyperlocomotion in mice. The administration of amphetamine increases dopaminergic and noradrenergic transmission in the central nervous system, causing hyperlocomotion in animals [31]. Similarly, the studied compound showed antipsychotic-like effects in the wide dose range (0.3–2.5 mg/kg). Significantly, the compound did not affect the locomotor activity of animals when given alone. 

Besides therapeutic effects in treating schizophrenia, the D_2_ receptor antagonists can induce extrapyramidal side effects. Thus, we assessed the risk of JJGW08 to induce catalepsy in mice. The catalepsy test evaluates the liability of potential antipsychotics to cause extrapyramidal side effects in humans. Catalepsy in rodents manifests itself as a state of muscle rigidity, where the animal fails to correct an externally imposed posture for a prolonged period. Our results demonstrated that JJGW08 showed cataleptogenic potential at a dose 67-fold higher than the lowest antipsychotic-like dose (10 mg/kg vs. 0.15 mg/kg). Thus, we might assume that the compound might have a low potential to induce extrapyramidal symptoms at antipsychotic-like doses. However, additional tests, especially after repeated administration of JJGW08, are needed to confirm this finding.

Central-acting drugs might impair motor coordination [32,33,34]. In addition, novel compounds that alter the neuromuscular coordination of animals may possess neurotoxic properties [35]. Therefore, we next determined the effect of JJGW08 on motor coordination in mice. Our studies revealed that JJGW08 did not induce motor coordination impairments at antipsychotic-like doses. The TD_50_ value was 121-fold higher than the lowest antipsychotic-like dose. However, more studies are needed to evaluate the compound’s central nervous system safety. 

Patients with schizophrenia often suffer from anxiety. Anxiety occurs in 30–60% of people with schizophrenia, affecting their quality of life and social interactions, as well as increasing negative self-esteem [36,37]. In addition, constant feelings of worry, nervousness, and uncertainty may be connected with the unpredictable appearance of positive and negative symptoms of schizophrenia [38,39]. Therefore, as the next step of our studies, we assessed the anxiolytic-like properties of JJGW08. We utilized the four-plate test, a commonly used screening test to evaluate the anxiolytic-like properties of the compounds. The four-plate test involves the suppression of the locomotor activity of an animal when it is given a mild electric foot shock as it moves from one quadrant of a metal-floored arena to another. Our studies revealed that JJGW08 showed an anxiolytic-like effect in the four-plate test in mice. Notably, JJGW08 did not affect locomotor activity in a 1-min session, which suggests that the observed effect is specific to the anxiolytic-like effect. Moreover, the compound showed an anxiolytic-like effect at a lower dose than diazepam, described in our previous studies [16]. Interestingly we observed an inverted-U-shaped dose effect (middle doses were effective, i.e., 0.3 and 0.625 mg/kg) of JJGW08 in this test. Such a non-linear dose-response is frequent in neuropharmacology and is difficult to explain due to its multifactorial nature. It might be due to the varying receptor occupancy at various doses, as JJGW08 is a multimodal compound targeting serotonin and dopamine receptors. Another explanation is the sedative effect of the compound that might hinder the anxiolytic-like properties at higher doses. However, this issue requires further studies.

We next assessed the anxiolytic-like properties of JJGW08 in the marble burying test to confirm the above findings. Our results confirmed the anxiolytic-like activity of JJGW08 observed in the four-plate test. However, the effect of JJGW08 in the marble burying test was visible at higher doses (i.e., 1.25 and 2.5 mg/kg), and we did not observe an inverted U-shaped dose effect. Since some antipsychotics show anxiolytic-like activity, we decided to compare the effects of JJGW08 in this test with aripiprazole, an atypical antipsychotic. Our experiments demonstrated that the effect of JJGW08 was comparable to the effect of a reference compound, aripiprazole, in this test. Interestingly, an analysis by Katzman indicates that because of the unique mechanism of action and safety profile, the use of aripiprazole for anxiety is an intriguing avenue of exploration [40]. Thus, the results of our study encourage further studies on JJGW08 and its possible use in anxiety.

Finally, to confirm the antipsychotic-like properties of JJGW08, we assessed the effect of the compound on MK-801-induced hyperlocomotion in rats. Similar to mice, JJGW08 showed an antipsychotic-like effect in a wide dose range (1–30 mg/kg) in rats, which confirmed the results obtained in mice. The reference compound, clozapine, also showed an antipsychotic-like effect in this test. Confirming the antipsychotic-like activity of JJGW08 in another species is a premise for further research on this compound.

Finally, we decided to test JJGW08 in rats’ prepulse inhibition (startle reduction) test. Startle reduction is a phenomenon in which a weak stimulus (prepulse) can suppress the startle response to a subsequent stronger startle stimulus (pulse). Clinical studies demonstrated that patients with schizophrenia, in particular, have deficits of prepulse inhibition. Our studies showed that JJGW08 did not reverse prepulse inhibition deficits in rats and thus did not show an antipsychotic-like effect. The available literature data show the ambiguous efficacy of atypical neuroleptics in reversing MK-801-induced sensorimotor gating deficits and, at the same time, show the lack of effect of classical neuroleptics [41,42]. A study by Bubeníková et al. demonstrated that not all atypical antipsychotics are effective in this test, i.e., olanzapine and clozapine restored MK-801-induced deficits in the prepulse inhibition test, but zotepine and risperidone were ineffective [43]. Moreover, the possible differences in the efficacy of atypical drugs may be related to the species diversity of the tested animal strains [41]. Scientists are also paying particular attention to the induction of schizophrenic-like disorders by MK-801, which somewhat reflects disease abnormalities in several different limbic regions [44]. Thus, in the case of JJGW08, the lack of confirmation of antipsychotic-like activity in the prepulse inhibition test in rats may be due to the complex activity of MK-801 and the blockade of NMDA receptors in various regions of the brain [44]. However, this issue requires more extensive analysis.

Limitations to our study include assessing the antipsychotic- and anxiolytic-like effects of JJGW08 only after a single administration. Chronic administration of the tested compound in the schizophrenia model would provide information on JJGW08 safety and long-term pharmacological effects. Moreover, we should also assess the affinity of the studied compound for a full panel of dopamine receptors, as they play an essential role in schizophrenia. Our future studies aim to evaluate the extensive pharmacological profile of JJGW08. 

## 4. Materials and Methods

### 4.1. Drugs

The studied compound 2-{5-[4-(2-metoksyfenylo)piperazin-1-ylo]pentoxy}benzamide hydrochloride, JJGW08, was synthesized in the Institute of Organic Chemistry and Technology, Faculty of Chemical and Engineering and Technology, Cracow University of Technology. The synthesis and biological properties of the compound were described earlier [18,19].

JJGW08 was dissolved in saline (0.9% NaCl, Polpharma, Starogard Gdańsk, Poland) and administered intraperitoneally (*ip)* 30 min before each behavioral test. The chemicals used in functional assays, i.e., serotonin (Sigma-Aldrich, Darmstadt, Germany), mianserin (Tocris Bioscience, Minneapolis, MN, USA), or methiotepin (Sigma-Aldrich, Darmstadt, Germany) were dissolved in DMSO (dimethyl sulfoxide, Sigma-Aldrich, Darmstadt, Germany). MK-801 (Sigma-Aldrich, Darmstadt, Germany) was dissolved in saline and administered *ip* 15 min before experiments, whereas amphetamine (Sigma-Aldrich, Darmstadt, Germany) was dissolved in saline and administered subcutaneously (*sc*) 30 min before tests. Olanzapine (Sigma-Aldrich, Darmstadt, Germany), aripiprazole (Sigma-Aldrich, Darmstadt, Germany), and clozapine (Sigma-Aldrich, Darmstadt, Germany) were dissolved in 1% Tween (J.T.Baker, Phillipsburg, NJ, USA) and administered *ip* 30 or 60 min before experiments. The control groups received saline or 1% Tween as a vehicle. JJGW08 was administered at a dose range of 0.08–2.5 mg/kg in mice and 0.3–30 mg/kg in rats.

### 4.2. Animals

Adult male Albino-Swiss mice (CD-1, 8 weeks old, 18–21 g) were obtained from an accredited house at the Faculty of Pharmacy, Jagiellonian University Medical College, Krakow, Poland, whereas male Wistar (WU) rats weighing 170–250 g were obtained from Mossakowski Medical Research Institute, Polish Academy of Sciences, Warsaw, Poland, and used for experiments. Animals were kept in groups of 10 mice or 4 rats in plastic standard cages (37 cm × 21 cm × 15 cm) in a controlled environment (i.e., constant room temperature (22 ± 2 °C), adequate humidity (40–60%), 12h light/dark cycle), with ad libitum access to food and water. Behavioral procedures were performed between 8 a.m. and 4 p.m. by a trained observer blind to the treatments. Animals were selected randomly for the treatment groups. Each group consisted of 8–10 mice or 6–8 rats that were used only once in each test. All injections were administered in a 10 mL/kg volume in mice and 1–2 mL/kg in rats. The animals were euthanized immediately after experiments. Procedures involving animals were conducted according to current European Community and Polish legislation on animal experimentation.

### 4.3. Functional Assay for 5-HT_1A_, 5-HT_2A_ and D_2_ Receptor

Tested and reference compounds were dissolved in dimethyl sulfoxide (DMSO) at a concentration of 10 mM. Serial dilutions were prepared in a 96-well microplate in assay buffer, and 8 to 10 concentrations were tested. Intrinsic activity assay was performed according to the manufacturer of the ready-to-use CHO-K1 cells with stable expression of the human serotonin 5-HT_1A_, 5-HT_2A,_ and D_2_ receptor, human GPCR, and the promiscuous G protein Gαqi/5 for D_2_ receptor and α_16_ for 5-HT_1A_ and 5-HT_2A_ (Perkin Elmer, Waltham, NA, USA). The assay was executed according to the previously described protocol [45]. After thawing, cells were transferred to assay buffer (DMEM/HAM’s F12 with 0.1% protease-free BSA) and centrifuged. The cell pellet was resuspended in assay buffer, and coelenterazine h was added at final concentrations of 5 µM. The cell suspension was incubated at 16 °C (or 21 °C), protected from light with constant agitation for 16 h (or 4 h), and then diluted with assay buffer to the concentration of 100,000 cells/mL (or 250,000 cells/mL). After 1 h of incubation, 50 µL of the cell’s suspension was dispensed using automatic injectors built into the radiometric and luminescence plate counter MicroBeta2 LumiJET (PerkinElmer, Waltham, NA, USA) into white opaque 96-well microplates preloaded with test compounds. Immediate light emission generated following calcium mobilization was recorded for 30 s. In antagonist mode, after 25–30 min of incubation, the reference agonist was added to the above assay mix, and light emission was re-recorded. The final concentration of the reference agonist (100 nM serotonin for the 5-HT_1A_ receptor, 30 nM α-methylserotonin for the 5-HT_2A_ receptor, and 30 nM apomorphine for the D_2_ receptor) was equal to EC_80_. IC_50_ and EC_50_ values were calculated.

### 4.4. Functional Assays for 5-HT_7_ Receptors

Test and reference compounds were dissolved in DMSO at a concentration of 1 mM. Serial dilutions were prepared in a 96-well microplate in assay buffer, and 8 to 10 concentrations were tested. For the 5-HT_7_ receptor, adenylyl cyclase activity was monitored using cryopreserved CHO-K1 cells expressing the human serotonin 5-HT_7_ receptor. A functional assay based on cells expressing the human 5-HT_7_ receptor was performed according to the previously described protocol [16,45]. CHO-K1 cells were transfected with a beta-lactamase (bla) reporter gene under the control of the cyclic AMP response element (CRE) (Life Technologies, Carlsbad, CA, USA).

Thawed cells were resuspended in stimulation buffer (HBSS, 5 mM HEPES, 0.5 IBMX, and 0.1% BSA at pH 7.4) at 200,000 cells/mL. The same cell suspension volume (10 μL) was added to tested compounds loaded onto a white opaque half-area 96-well microplate. The antagonist response experiment was performed with 10 nM serotonin as the reference agonist. The agonist and antagonist were added simultaneously. Cell stimulation was performed for 60 min at room temperature. After incubation, cAMP measurements were performed with homogeneous TR-FRET immunoassay using the LANCE Ultra cAMP kit (PerkinElmer, Waltham, MA, USA). Then, 10 μL of EucAMP Tracer Working Solution and 10 μL of ULight-anti-cAMP Tracer Working Solution were added, mixed, and incubated for 1 h. The TR-FRET signal was read on an EnVision microplate reader (PerkinElmer, Waltham, NA, USA). IC_50_ and EC_50_ values were calculated by non-linear regression analysis using GraphPad Prism 5.0 software. The log IC_50_ was used to obtain the K_b_ by applying the Cheng–Prusoff approximation.

### 4.5. MK-801-and Amphetamine-Induced Hyperlocomotion Test in Mice

The test was performed according to the method described by Carlsson et al. [46,47]. The mobility of the animals was measured in actometers, i.e., in plastic Opto M3 cages (22 × 12 × 13 cm) connected to a computer with MultiDevice Software v.1.30 (Columbus Instruments, Columbus, OH, USA). The experimental cages were equipped with infrared sources on one side and sensors receiving the emitted rays on the other side of the cage. The crossing of each beam of infrared rays was classified as motor activity. The animals were placed individually in experimental cages immediately after administration of the tested compound, 30 min before the start of the test, to adapt to the new conditions and exclude the occurrence of hyperactivity caused by the change of the environment. Spontaneous locomotor activity was measured every 5 min for 60 min. Mice received two injections: of the tested compound or reference substance (30 min before the test, *ip*) and of amphetamine (2.5 mg/kg, 30 min before the test, *sc*) or MK-801 (0.2 mg/kg, 15 min before the test, *ip*). Control groups received an injection of saline and amphetamine or MK-801, or two injections of saline, respectively. Olanzapine was used as a reference compound. 

### 4.6. Catalepsy Bar Test

Catalepsy was assessed using the bar method described by Ueki et al. with minor modifications [48,49]. The front paws of the mice were placed on a cylindrical metal bar located 4 cm above the tabletop’s surface, while the hind paws remained on the tabletop. The time in which the animal held both paws on the bar was measured at 30, 60, and 120 min after the administration of the tested compound, with a maximum measurement time of 60 s. Each of the measurements consisted of placing the animal’s paws on the bar three times unless the mouse was on it for 60 s; then, no further trial was performed. The score for each trial was assessed as follows [21]:

- 0 points if the animals held the constrained position < 15 s;
- 1 point when the animal stayed on the bar for 15–29.9 s;
- 2 points for when the animal stayed on the bar for 30–59.9 s;
- 3 points for staying on the bar for more than 60 s.

The minimum cataleptogenic dose was defined as the lowest dose inducing a mean catalepsy score of ≥1 at 30, 60, or 120 min post-treatment. The tested compound was administered *ip* at the dose range of 5–20 mg/kg.

### 4.7. Rotarod Test

The experimental procedure was described in detail by Pytka K. et al. [50]. Mice were trained on the rotarod apparatus (May Commat RR0711, Ankara, Turkey; rod diameter: 2 cm) for 3 consecutive days. During each training session, animals were placed for 3 min on the rotating rod (24 rpm, constant speed) with unlimited trials. The experiment was performed 24 h after the last training session. On the test day, mice were injected with studied compounds and, 30 min later, placed on the rotarod. The criterion of motor impairments was the inability of the animal to remain on the rotating rod for 60 s. The TD_50_ value was calculated as a dose at which 50% of the animals could not stay on the rotating rod [22].

### 4.8. Four-Plate Test

The four-plate test was performed on mice according to the previously described method [51,52]. Mice were placed individually in the four-plate apparatus (Panlab, Barcelona, Spain). After a 15 s habituation period, each mouse that crossed from one plate to another (two limbs on one plate, two on another) was punished by an electric shock (0.8 mA, 0.5 s). The number of punished crossings was calculated during the 60 s of the test. 

### 4.9. Marble Burying Test

The test was conducted according to the method described by Broekkamp et al. [53] with minor modifications. Mice were placed individually in plastic cages, identical to their home cages, that contained a layer of bedding and 20 glass balls (1.6 cm in diameter) arranged in a pattern 4 × 5. After 30 min of the experiment, the mice were removed from the cages, and the number of balls buried to at least 2/3 of their size was counted. The reduction in the number of buried balls compared to the control group suggests the tested compound’s anxiolytic-like properties. 

### 4.10. Spontaneous Locomotor Activity in Mice

The locomotor activity of mice was measured as previously described [16,54] using the same apparatus as described in paragraph 4.5. Each mouse was placed individually in a cage for a 30 min habituation period (directly after administration of the studied compound), and then the number of photobeams crossings was recorded (ambulation). Locomotor activity was evaluated every 1 or 5 min for 1–60 min depending on the observation period in behavioral tests (60 min for the hyperlocomotion test, 30 min for the marble burying test, and 1 min for the four-plate test). The cages were disinfected with an odorless disinfection solution after each mouse.

### 4.11. Open Field Test after MK-801 Administration in Rats

The experiment was evaluated according to the method described earlier [55,56]. It was performed in a soundproof chamber with a 30-min habituation period 24 h before the procedure. The open field apparatus consisted of four black, octagonal, stainless steel arenas (diameter: 80 cm) with 30 cm high walls. Each arena was divided into a peripheral and central sector. The peripheral sector was defined as the region within 20 cm from the walls. The approximate light intensity was 15 lx in the central point of the arena and 4 lx close to its walls. 

Individual rats were placed in the central part of the open field and allowed to explore the whole arena for 30 min under dim light (approximately 10 lx) and continuous white noise (65 dB). Forward locomotion (cm/30 min) was registered in 10-min intervals for 30 min and analyzed with the aid of a computerized video tracking system (Videomot, TSE, Bad Homburg, Germany) [57,58]. The arenas were cleaned between tests with 20% (v/v) alcohol and allowed to dry.

The tested compound was administered *ip* 30 min before the experiment at the dose range of 0.3–30.0 mg/kg, and MK-801 (0.3 mg/kg,) was administered *ip* 15 min before the procedure. The reduction of MK-801-induced hyperactivity of animals is thought to reflect the antipsychotic-like effect of the tested compound [59]. Clozapine (3.0–30.0 mg/kg, *ip*), used as a reference drug, was administered 60 min before the experiment.

### 4.12. Prepulse Inhibition Test in Rats

The study was conducted as described in detail by Acewicz and colleagues [60]. The PPI apparatus consisted of eight identical startle chambers (SR-LAB, San Diego Instruments, San Diego, CA, USA). Each chamber consisted of a Plexiglas cylinder (8.8 cm diameter × 18.4 cm length) resting on a Plexiglas frame located in a sound-attenuated and ventilated enclosure. Background noise and acoustic stimuli were presented via a loudspeaker mounted 24 cm above the cylinder. Startle responses, reflecting the motion of animals in the cylinder following the acoustic stimulus, were detected by a piezoelectric transducer mounted below the frame. The administration of stimuli and response recording were controlled by the SR-LAB software. Sound levels in the chambers were measured and calibrated with a sound meter. Response sensitivities were calibrated using the SR-LABStartle Calibration System. A chamber light was on throughout the session, and the white background noise was set at 70 dB. Each session started with a 5-min acclimatization period to accustom the rat to the experimental procedure and lasted 30 min. The rats were placed individually in the Plexiglas cylinder. Three startling stimuli (120 dB, duration: 40 ms) were given during the acclimatization period with an average inter-trial interval (ITI) of 22.5 s (15–30 s). The SR-LAB software randomized the ITI. The initial stimuli were followed by test trials presented in random order. The PPI session involved: 10 trials with a sham stimulus (70 dB, 40 ms), 10 prepulse trials (PP) which included only 20-ms PP stimuli (84 dB or 90 dB), 10 pulse trials (P) which included only a pulse (startling) stimulus (120 dB, 40 ms), and 10 prepulse-and-pulse trials (PP-P) which included a 20-ms PP (84 dB or 90 dB) followed 100 ms later by a 40-ms P stimulus (120 dB). The mean ITI was 22.5 s. Startle responses were measured for 100 ms after the onset of the last stimulus within each trial. Startle amplitudes were averaged across 10 trials for each type of test trial. The magnitude of the PPI was calculated as a percent inhibition of the startle amplitude in the P trial (treated as 100%) according to the formula: 

[(startle amplitude in P trials–startle amplitude in PP-P trials)/startle amplitude in P trials] × 100%.

((PA − PPA)/PA) × 100%,(1)

Startle responses to the three initial stimuli were excluded from the statistical analyses. Reversing the sensorimotor gating deficits induced by MK-801 may suggest the potential antipsychotic effect of the tested compound. The studied compound at the doses 3.0–30 mg/kg was administered *ip* 30 min before the experiment, whereas MK-801 at the dose of 0.6 mg/kg was administered *ip* 15 min before the procedure.

### 4.13. Statistical Analysis

Results are presented as means ± SD (standard deviation). Comparisons between experimental and control groups were performed by one- or two-way ANOVA, followed by Newman–Keuls or Bonferroni *post hoc,* respectively. In cases when assumptions for normal distribution of data was not fulfilled (determined using the D’Agostino and Pearson test), we used Kruskal–Wallis with Dunn’s *post hoc* test.

## 5. Conclusions

In this study, we found that JJGW08, a novel arylpiperazine alkyl derivative of salicylamide, possessed strong antagonistic properties at dopamine D_2_ and serotonin 5-HT_1A_ and very weak at serotonin 5-HT_2A_ and 5-HT_7_ receptors. Furthermore, the compound showed antipsychotic- and anxiolytic-like properties in rodents. JJGW08 did not affect locomotor coordination or induce catalepsy in mice at antipsychotic-like doses. Therefore, our study suggests JJGW08 could be a model structure for synthesizing new arylpiperazine alkyl derivatives of salicylamide with potential use in treating schizophrenia with anxiety.

## Figures and Tables

**Table 1:** The intrinsic activity of JJGW08 for serotonin 5-HT_1A_, 5-HT_2A_, and 5-HT_7_ receptors and dopamine D_2_ receptors.

Receptor	Treatment	Agonist Mode		Antagonist Mode			
		E_max_%	*p*EC_50_ ± Range	E_max_%	*p*IC_50_ ± Range	K_b_ [nM]	R^2^K_b_
5-HT_1A_	Serotonin	100	7.6 ± 1.1	0	n.c.	n.c.	n.c.
	NAN-190	6	n.c.	0	9.0 ± 0.1	0	0.99
	JJGW08	9	n.c.	1	8.3 ± 0.1	0	0.98
5-HT_2A_	α-methylserotonin	100	8.5 ± 0.3	2	n.c.	n.c.	n.c.
	Serotonin	112	8.4 ± 0.0	1	n.c.	n.c.	n.c.
	Mianserin	3	n.c.	3	8.1 ± 0.1	2	0.91
	JJGW08	21	5.9 ± 0.1	5	5.7 ± 0.4	290	0.93
5-HT_7_	Serotonin	100	8.1 ± 0.1	0	n.c.	n.c.	n.c.
	SB-269970	0	n.c.	9	9.3 ± 0.2	0.2	0.94
	JJGW08	1	n.c.	4	6.4 ± 0.1	190	0.97
D_2_	Quiniprole	100	8.7 ± 0.1	0	n.c.	n.c.	n.c.
	Apomorphine	100	7.5 ± 0.1	0	n.c.	n.c.	n.c.
	Chlorpromazine	2	n.c.	0	9.8 ± 0.4	0	0.94
	JJGW08	13	n.c.	0	7.9 ± 0.0	2	0.97

Data are expressed as the mean ± range of two independent experiments in duplicate. E_max_—the maximum possible effect; *p*EC_50_—the negative logarithm of the concentration of a compound where 50% of its maximal effect was observed, which was divided by the standard concentration (K_std_ = 1 M); *p*IC_50_—the negative logarithm of the concentration of a compound where 50% of its maximal inhibitory effect was observed, which was divided by the standard concentration (K_std_ = 1 M); K_b_—the equilibrium dissociation constant of a competitive antagonist determined using of the Cheng equation [20]; R^2^—the coefficient of determination; n.c.—non-calculable.

**Table 2:** The evaluation of cataleptogenic properties of JJGW08.

Treatment	Dose (mg/kg)		Mean Score	
		30 min	60 min	120 min
	5	0.0	0.2	0.2
JJGW08	10	0.7	0.9	1.4
	20	2.1	2.1	1.5
	5	0.8	0.8	0.7
Olanzapine	10	0.7	1.6	1.3
	20	0.7	2.0	1.8

Mice were placed on a cylindrical metal bar above the tabletop’s surface, while the hind paws remained on the tabletop. The time in which the animal held both paws on the bar was measured at 30, 60, and 120 min after the intraperitoneal (*ip*) administration of JJGW08, with a maximum measurement time of 60 s. Data are presented as the mean score for each trial, which was assessed according to Ögren et al. [21]. The minimum cataleptogenic dose was defined as the lowest dose inducing a mean catalepsy score of ≥1 at 30, 60, or 120 min post-treatment. *n* = 10 mice per group.

**Table 3:** The effect of JJGW08 on motor coordination in mice.

Treatment	Dose (mg/kg)	Animals That Fell from Rotating Rod	Time before Animals Fell (s)	TD_50_ (mg/kg)
JJGW08	10	2/8	58 ± 4	18 (11–29)
	20	4/8	41 ± 24	
	30	6/8	20 ± 25	

Mice previously trained for 3 consecutive days were placed individually on a rotating rod for 60 s. The time remaining on the rod was recorded. The TD_50_ value [22] was calculated as a dose at which 50% of the animals could not stay on the rotating rod. JJGW08 was administered intraperitoneally (*ip*) 30 min before the test. Values are expressed as means ± SD, *n* = 8 mice per group.

**Table 4:** The influence of JJGW08 on locomotor activity in mice.

Treatment	Dose (mg/kg)	Number of Crossings ± SD								
		60 min			30 min			1 min		
Saline	-	1140	±	600	866	±	653	45	±	21
	0.15	1346	±	886	547	±	343	38	±	15
	0.3	1588	±	772	1027	±	733	46	±	21
JJGW08	0.625	1671	±	923	634	±	716	27	±	14
	1.25	1769	±	835	1181	±	732	22	±	14
	2.5	1450	±	1252	437	±	588	19	±	24

Locomotor activity was recorded separately for each mouse in actometers. After the 30-min adaptation period, the number of photobeams crossings was measured at the appropriate time intervals, i.e., 60 min for the hyperlocomotion test, 30 min for the marble burying test, and 1 min for the four-plate test. JJGW08 was administered intraperitoneally (*ip*) 30 min before the test. The control group received an injection of 0.9% NaCl (*ip*). Values are expressed as means ± SD, n = 8–10 mice per group. Statistical analysis: one-way ANOVA (Newman–Keuls *post hoc*).

## Data Availability

Data are contained within the article.

## Supplementary Materials

The following supporting information can be downloaded at: https://www.mdpi.com/article/10.3390/ijms232415929/s1.
