# Supplementary material for: Antipsychotic- and Anxiolytic-like Properties of a Multimodal Compound JJGW08 in Rodents
Source: Int J Mol Sci. 2022 Dec 14;23(24):15929. doi: 10.3390/ijms232415929 (PMC9781916; doi:10.3390/ijms232415929)

Supplementary data

Figure S1. Functional analysis to 5-HT_{1A} receptor.

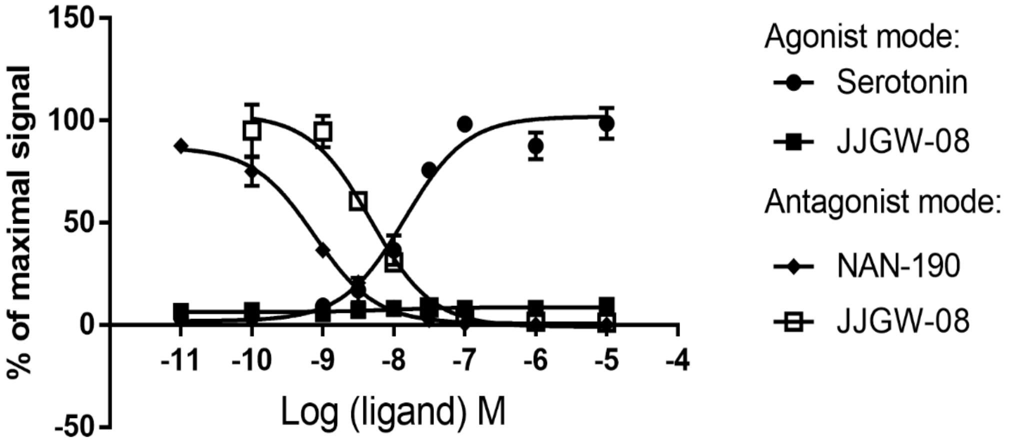

Figure S2. Functional analysis to 5-HT_{2A} receptor.

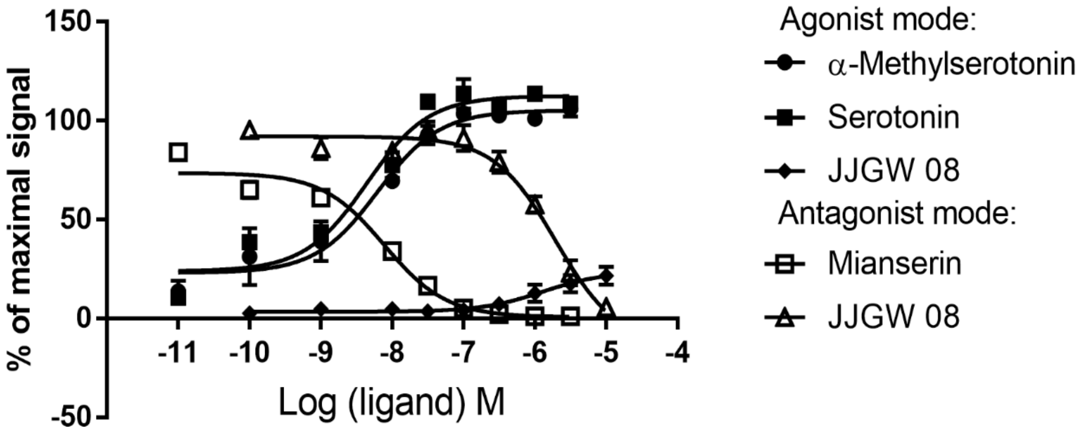

Figure S3. Functional analysis to 5-HT_7 receptor.

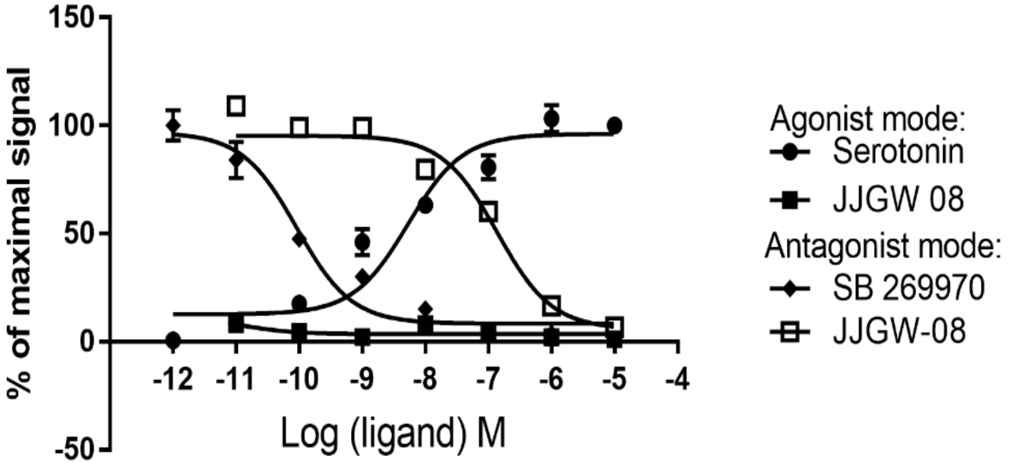

Figure S4. Functional analysis to D_2 receptor.

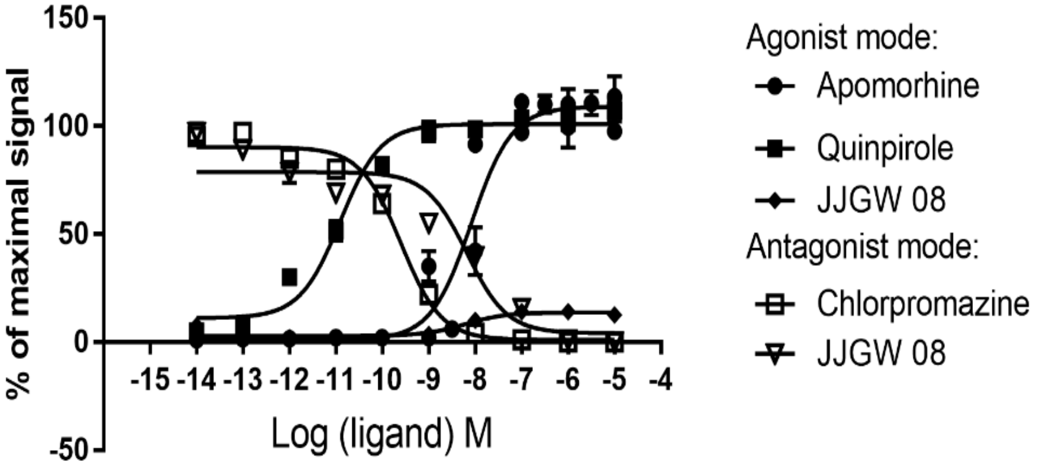

Supplement: Supplementary file 1 [file ijms-23-15929-s001.zip › ijms-2030902-supplementary.pdf]
